# Confidence response times: Challenging postdecisional models of confidence

**DOI:** 10.1167/jov.23.7.11

**Published:** 2023-07-14

**Authors:** Sixing Chen, Dobromir Rahnev

**Affiliations:** 1 School of Psychological and Cognitive Sciences, Peking University, Beijing, China; 2 School of Psychology, Georgia Institute of Technology, Atlanta, GA, USA

**Keywords:** confidence response time, confidence computation, perceptual decision-making, metacognition

## Abstract

Even though the nature of confidence computations has been the topic of intense interest, little attention has been paid to what confidence response times (cRTs) reveal about the underlying confidence computations. Several previous studies found cRTs to be negatively correlated with confidence in the group as a whole and consequently hypothesized the existence of an intrinsic relationship of cRT with confidence for all subjects. This hypothesis was further used to support postdecisional models of confidence that predict that cRT and confidence should always be negatively correlated. Here we test the alternative hypothesis that cRT is driven by the frequency of confidence responses such that the most frequent confidence ratings are inherently made faster regardless of whether they are high or low. We examined cRTs in three large data sets from the Confidence Database and found that the lowest cRTs occurred for the most frequent confidence rating. In other words, subjects who gave high confidence ratings most frequently had negative confidence–cRT relationships, whereas subjects who gave low confidence ratings most frequently had positive confidence–cRT relationships. In addition, we found a strong across-subject correlation between response time and cRT, suggesting that response speed for both the decision and the confidence rating is influenced by a common factor. Our results show that cRT is not intrinsically linked to confidence and strongly challenge several postdecisional models of confidence.

## Introduction

Humans have the metacognitive ability to estimate the accuracy of their decisions (Metcalfe & Shimamura, 1994), which can guide their learning and subsequent actions (Desender et al., 2018; Fleming et al., 2012; Nelson & Narens, 1990; Shimamura, 2000; Yeung & Summerfield, 2012). However, how one computes a confidence estimate for a particular decision remains poorly understood despite the fact that confidence computations have been a topic of intense interest in metacognition research (Rahnev et al., 2022).

One potentially promising but little-explored avenue toward understanding confidence computations is the examination of confidence response times (cRTs). Previous research found cRT to be associated with confidence and decision accuracy (Baranski & Petrusic, 1998; Herregods et al., 2023; Moran et al., 2015; Pleskac & Busemeyer, 2010). Specifically, these studies have claimed that confidence ratings are computed faster whenever people are more confident or more accurate. These relationships were further interpreted as evidence that confidence is based on a postdecision evidence accumulation process (Herregods et al., 2023; Moran et al., 2015; Pleskac & Busemeyer, 2010; Yu et al., 2015). Postdecision evidence accumulation models assume that confidence is necessarily based on additional evidence accumulated after the decision is made. For example, in the two-stage dynamic signal detection (2DSD) optional stopping model (Pleskac & Busemeyer, 2010), different confidence levels have different confidence boundaries. The 2DSD optional stopping model assumes that every time the evidence crosses a confidence boundary, there is a certain probability that the accumulation process will be terminated and a corresponding confidence response will be made. Another two models (Herregods et al., 2023; Moran et al., 2015) assume the existence of collapsing confidence boundaries that ensure that higher confidence responses are given faster than lower confidence responses. Thus, substantial theoretical claims have been made based on the relationship of cRT with confidence and accuracy.

**Figure 1.**
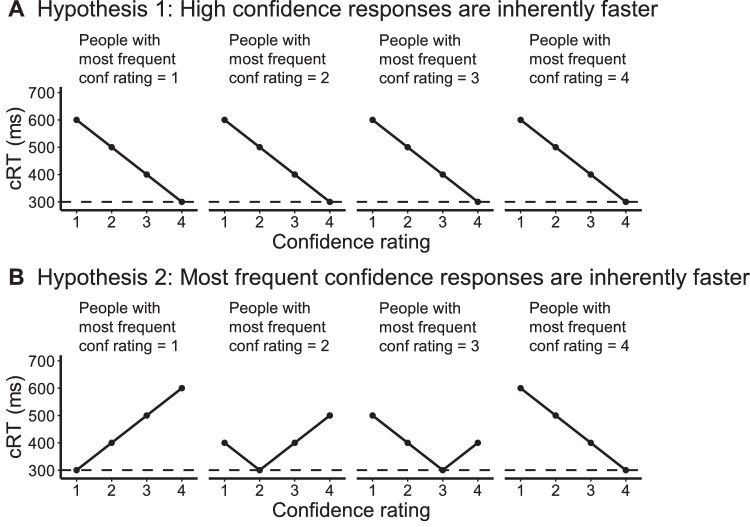
Illustration of the two hypotheses regarding the relationship between cRT and confidence. (**A**) Hypothesis 1 predicts that high-confidence responses are inherently made faster regardless of which confidence response is the most frequent. Therefore, the same decrease in cRT should be observed for all subjects. (**B**) Hypothesis 2 predicts that the most frequent confidence responses are inherently made faster regardless of whether they are high or low. Therefore, the relationship between cRT and confidence ratings would be different across subjects based on each subject's confidence bias.

The crucial hypothesis underlying postdecisional evidence accumulation models is that high-confidence responses are inherently made faster (Hypothesis 1; Figure 1A). However, a previously unexamined alternative hypothesis is that cRT is driven by the frequency of confidence responses such that the most frequent confidence ratings are inherently made faster regardless of whether they are high or low (Hypothesis 2; Figure 1B). This hypothesis is motivated by extensive literature showing that more frequent motor actions are executed faster (Katzner & Miller, 2012; Mattes et al., 2002; Miller, 1998; Näätänen, 1971). Hypothesis 2 thus predicts that for subjects who are biased toward low confidence, cRT will be lower for their low- versus high-confidence ratings but that the opposite relationship would be seen for subjects biased toward high confidence. In other words, according to Hypothesis 2, there is no intrinsic cRT–confidence relationship, and instead, any observed relationship is due to subjects responding faster for their most frequent confidence ratings.

Here we adjudicated between the two hypotheses about the cRT–confidence relationship. To do so, we analyzed three large data sets from the Confidence Database (Rahnev et al., 2020) and examined whether the pattern of results matched the predictions of Hypothesis 1 or Hypothesis 2. The results followed closely the predictions of Hypothesis 2, thus strongly challenging the assumed intrinsic relationship between cRT and confidence (Hypothesis 1). These results cast doubt on models that feature postdecision evidence accumulation processes that necessarily result in a negative cRT–confidence relationship.

## Methods

### Data set selection

To adjudicate between the two hypotheses above, we sought to examine the relationship of cRT with confidence and accuracy in data sets with large sample sizes. Specifically, we searched for data sets that (a) included confidence ratings with up to 4-point scales, (b) recorded cRTs, and (c) had at least 75 subjects who each completed at least 200 trials per task. Note that we selected data sets with discrete confidence scales with less than or equal to four confidence levels because we analyzed separately groups of subjects based on their most frequent confidence response, and having more detailed confidence scales leads to too many subgroups that diminish in sample size. We searched the 171 data sets included in the Confidence Database (Rahnev et al., 2020) as of December 1, 2022, and found three data sets that met the above conditions: “Bang_2019_Exp2,” “Haddara_2022_Expt1,” and “Haddara_2022_Expt2.” For simplicity, here we call these data sets “Bang,” “Haddara1,” and “Haddara2,” respectively. In addition, to further examine the robustness of our results, we relaxed the third criterion so that data sets with at least 30 (instead of 75) subjects who each completed 150 (instead of 200) trials per task would be selected. These more liberal selection criteria resulted in the selection of three additional data sets (“Maniscalco_2017_expt1,” “Maniscalco_2017_expt2,” and “Yeon_unpub_Exp2”; Supplementary Methods). Analyses of these data sets led to the same conclusions (Supplementary Figures S1–S3).

### Experimental designs

Complete details about the experiments can be found in the original articles (Bang et al., 2019; Haddara & Rahnev, 2022). All data sets featured two-choice perceptual decisions with 4-point confidence ratings given with separate button presses. The decisions and confidence ratings were untimed and given with a computer keyboard. Decisions were given with keys “1” and “2.” Confidence ratings were given with keys “1,” “2,” “3,” and “4,” with “1” indicating lowest confidence and “4” indicating highest confidence. Below, we provide a bit more detail regarding each of the three data sets.

**Figure 2.**
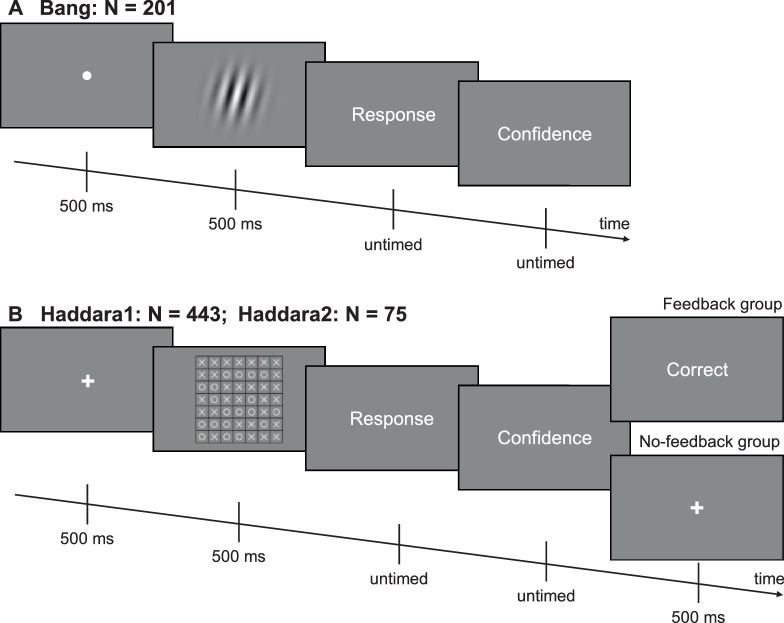
Experimental tasks. (**A**) The experimental task in the Bang data set. Subjects indicated whether a Gabor patch was tilted clockwise or counterclockwise from vertical. The data set consists of coarse discrimination and fine discrimination tasks with the contrasts and tilt angles of the Gabor patches varying between the two tasks. The Gabor patch shown here is only for an illustration purpose and does not faithfully represent stimuli in either of the two tasks. (**B**) The experimental task in the Haddara1 and the Haddara2 data sets. In Haddara1, subjects saw a 7 × 7 grid that consisted of the letters X and O (Task 1) or the colors red or blue (Task 2) and indicated which letter or color occurred more frequently. (The illustration of Task 2 is not shown here.) Approximately half of the subjects received trial-by-trial feedback in Task 1, while no feedback was given in Task 2. The task design in Haddara2 is identical to Task 1 in Haddara1.

In the Bang data set (Bang et al., 2019), subjects (*N* = 201) indicated whether a Gabor patch was tilted clockwise or counterclockwise from vertical (Figure 2A). The data set consists of two tasks. For the coarse discrimination task, the Gabor patches were embedded in noise and tilted 45 degrees away from the vertical. For the fine discrimination task, the Gabor patches were tilted about 1 degree away from vertical. The contrast in the coarse discrimination task and the tilt in the fine discrimination task varied between subjects in order to match the average performance across the two tasks. Each subject completed 100 trials for each of the two tasks. Here we combined the data from both tasks.

In the Haddara1 data set (Haddara & Rahnev, 2022), subjects (*N* = 443) saw a 7 × 7 grid that consisted of the letters X and O (Task 1; Figure 2B) or the colors red or blue (Task 2). Subjects indicated which letter or color occurred more frequently. In Task 1, approximately half of the subjects received trial-by-trial feedback about whether the judgment was correct while the other half received no such feedback. No feedback was given in Task 2. The proportion of the dominant stimulus was 31 of 49 for Task 1 and 27 of 49 for Task 2. Each subject completed 330 trials for Task 1 and 150 trials for Task 2. Here we again combined the data from both tasks and analyzed together subjects who did or did not receive trial-by-trial feedback.

For the Haddara2 data set (Haddara & Rahnev, 2022), the task design was identical to Task 1 in Haddara1 (Figure 2B). A new sample of subjects (*N* = 75) completed seven sessions over 7 different days. Each subject completed 500 trials per day and 3,500 in total. Approximately half of the subjects received trial-by-trial feedback about whether the judgment was correct, while the other half received no such feedback. We again analyzed together subjects who did or did not receive trial-by-trial feedback. Note that even though Haddara1 and Haddara2 used the same task, these data sets featured different distributions of confidence biases. Because Haddara2 includes 7 days, it is possible that these differences are due to practice effects. To check for this possibility, we separately analyzed the data from day 1 of Haddara2 (Supplementary Figure S4).

### Analyses

For each subject in each of the three data sets, we excluded trials with response times (RTs) outside mean ± 3 × *SD*s or cRTs outside mean ± 3 × *SD*s before conducting any data analyses. We coded confidence ratings as scalar variables with values 1–4 when we used them for analyses.

We divided subjects into four different groups according to their most frequent confidence ratings and examined whether the cRT–confidence relationship varied between groups. To measure the cRT–confidence relationship, we performed linear regressions on cRT as a function of confidence for each subject and used the slopes of the regressions (β_cRT∼Confidence_) as an indicator of the cRT–confidence relationship for each individual. We performed linear regressions on β_cRT∼Confidence_ as a function of groups to test the effects of groups on the cRT–confidence relationship.

We then tested the cRT–confidence relationship at the population level across different data sets to examine whether the relationship is universal. To determine the effect of confidence on cRT at the population level, we performed linear mixed-effects model analyses on cRT as a function of confidence with random intercepts and random slopes on confidence between subjects and examined the fixed effects of confidence on cRT. Besides, we also tested the cRT–confidence relationship at the individual level (Supplementary Figure S5). We separately computed β_cRT∼Confidence_ in odd and even trials for each subject and correlated these values across subjects to test whether the individual differences are stable and consistent. For robustness, we also bootstrapped 100 random split-half partitions of trials for each subject and tested whether β_cRT∼Confidence_ is correlated between the two halves. We transformed *r* values of correlations to *z* scores, averaged *z* scores obtained from 100 partitions, and reported *r* values transformed from the averaged *z* scores.

In addition, we also examined the cRT–accuracy relationship. To measure the cRT–accuracy relationship, we computed the differences in cRT between correct and error trials (cRT_correct_ – cRT_error_) for each subject. We performed linear regressions on cRT_correct_ – cRT_error_ as a function of groups to test the effects of groups on the cRT–accuracy relationship and examined the cRT–accuracy relationship across different data sets. To determine the effect of accuracy on cRT at the population level, we performed paired-sample *t*-tests comparing cRT for correct and error trials. We also tested the cRT–accuracy relationship at the individual level by separately computing cRT_correct_ – cRT_error_ in odd and even trials for each subject and correlating these values across subjects (Supplementary Figure S6). We also bootstrapped 100 random split-half partitions of trials for each subject for cRT_correct_ – cRT_error_.

Finally, to further assess the extent a single factor drives the response speed for both the decision and the confidence rating, we computed the RT–cRT correlation across subjects in each data set.

All analyses were conducted in R software environment (Version 4.1.2). Bayes factors were computed with the R package “BayesFactor” (Version 0.9.12–4.4). Linear mixed-effects models were implemented with the R package “lmerTest” (Version 3.1.3).

### Data and code

All data and code are available at https://osf.io/n5f24.

## Results

We investigated the nature of the cRT–confidence relationship. Specifically, we adjudicated between the hypothesis that high-confidence responses are inherently made faster regardless of which confidence response is the most frequent (Hypothesis 1) and the hypothesis that the most frequent confidence responses are inherently made faster regardless of whether they are high or low (Hypothesis 2).

### cRT–confidence relationship

**Figure 3.**
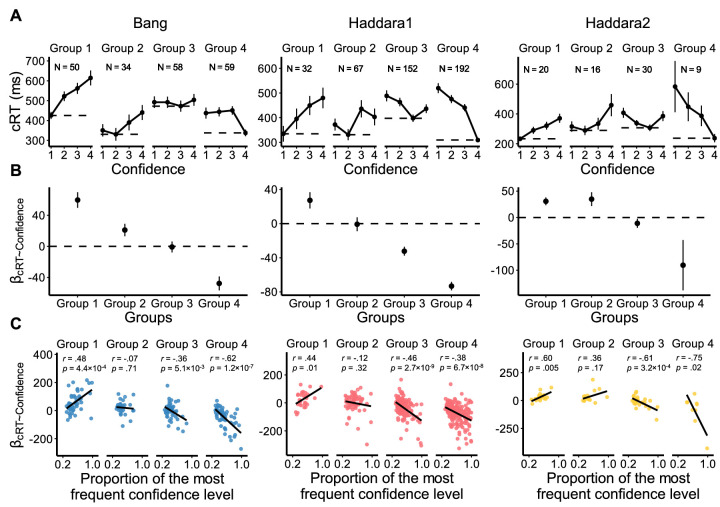
The cRT–confidence relationship is driven by the most frequently chosen confidence rating. (**A**) cRT for each possible confidence rating plotted separately for each group formed based on the most frequent confidence rating (e.g., “Group k” consists of all subjects for whom k is the most frequently chosen confidence rating). Horizontal dashed lines indicate the lowest cRTs among the four confidence levels in each group. (**B**) The cRT–confidence relationship, quantified as β_cRT∼Confidence_, for each of the groups formed based on the most frequent confidence rating. (**C**) The cRT–confidence relationship within each group depends on the proportion of trials on which a subject used the most frequent rating. In accordance with Hypothesis 2, we find positive relationships between β_cRT∼Confidence_ and the proportion of the most frequent confidence rating for Group 1 but negative relationships for Group 4. Error bars show *SEM*. Each dot corresponds to one subject. Solid lines indicate best-fitting regressions.

We first tested the predictions of Hypotheses 1 and 2 (see Figure 1). According to Hypothesis 2, cRTs should be lowest for the more frequently used confidence rating. If so, subjects who give low confidence most frequently should be fastest for low-confidence ratings and slowest for high-confidence ratings, whereas subjects who give high confidence most frequently should be fastest for high-confidence ratings and slowest for low-confidence ratings. Conversely, Hypothesis 1 predicts that all subjects should be fastest for high-confidence ratings and slowest for low-confidence ratings regardless of their most frequent confidence rating. To compare the predictions of the two hypotheses, we divided subjects into groups depending on their most frequent confidence ratings and examined which confidence rating was made the fastest in each group. Consistent with Hypothesis 2, the lowest cRTs always corresponded to the most frequent confidence levels in all four groups in each of the three data sets (probability of this happening by chance equals ${\left(\frac{1}{4}\right)}^{12}=6.0\;\times 10^{-8}$; Figure 3A).

Beyond examining the identity of the most frequent confidence rating, we also explored how the direction of the cRT–confidence relationship changed based on the most frequent confidence rating. Hypothesis 2 predicts that the direction of this relationship should switch from positive to negative for people who give low confidence versus high confidence most frequently. Conversely, Hypothesis 1 predicts that the direction of this relationship should always be negative regardless of which confidence rating is most frequent. For each of the three data sets, we found that subjects in Group 1 (who rated the lowest confidence level the most frequently) showed a significant positive cRT–confidence relationship (quantified as the slope β_cRT∼Confidence_) (Bang: *t*(49) = 5.94, *p* = 2.9 × 10^−7^, Cohen's *d* = .84, BF_10_ = 5.2 × 10^4^; Haddara1: *t*(31) = 2.87, *p* = 0.007, Cohen's *d* = .51, BF_10_ = 5.70; Haddara2: *t*(19) = 4.14, *p* = 5.6 × 10^−4^, Cohen's *d* = −.93, BF_10_ = 60.61; Figure 3B), while subjects in Group 4 (who rated the highest confidence level the most frequently) showed a negative cRT–confidence relationship (Bang: *t*(58) = −5.33, *p* = 1.7 × 10^−6^, Cohen's *d* = −.69, BF_10_ = 9.8 × 10^3^; Haddara1: *t*(190) = −14.75, *p* = 2.5 × 10^−33^, Cohen's *d* = −1.07, BF_10_ = 1.1 × 10^30^; Haddara2: *t*(8) = −1.90, *p* = 0.09, Cohen's *d* = −.63, BF_10_ = 1.14). Analyzing all groups together, we found that the slope of the cRT–confidence relationship (i.e., β_cRT∼Confidence_) decreased for the groups in which the most frequent confidence rating was higher (Bang: slope = −34.66, *t*(199) = −9.12, *p* = 8.4 × 10^−17^, Cohen's *d* = −.64; Haddara1: slope = −35.05, *t*(440) = −10.34, *p* = 1.3 × 10^−22^, Cohen's *d* = −.49; Haddara2: slope = −34.22, *t*(73) = −4.52, *p* = 2.4 × 10^−5^, Cohen's *d* = −.53; Figure 3B). These results strongly support Hypothesis 2 and demonstrate that the patterns in cRT results are largely determined by the identity of the most frequently chosen confidence rating.

Beyond the differences between groups, Hypothesis 2 makes another prediction about the variability expected within each group. Specifically, the effects within each group should depend on the frequency of the most frequent rating. For example, among subjects who rated confidence = 1 most frequently (i.e., Group 1), subjects with higher proportions of confidence = 1 responses should exhibit larger cRT–confidence slopes (β_cRT∼Confidence_), which is exactly what we found (Bang: *r* = .48, *p* = 4.4 × 10^−4^; Haddara1: *r* = .44, *p* = 0.01; Haddara2: *r* = .60, *p* = 0.005; Figure 3C). Conversely, among subjects who rated confidence = 4 most frequently (i.e., Group 4), subjects with higher proportions of confidence = 4 responses should exhibit smaller cRT–confidence slopes (β_cRT∼Confidence_), which is again what we found (Bang: *r* = −.62, *p* = 1.2 × 10^−7^; Haddara1: *r* = −.38, *p* = 6.7 × 10^−8^; Haddara2: *r* = −.75, *p* = 0.02). Therefore, Hypothesis 2 is further supported by these within-group analyses (note that Hypothesis 1 predicts no such correlations for any group).

**Figure 4.**
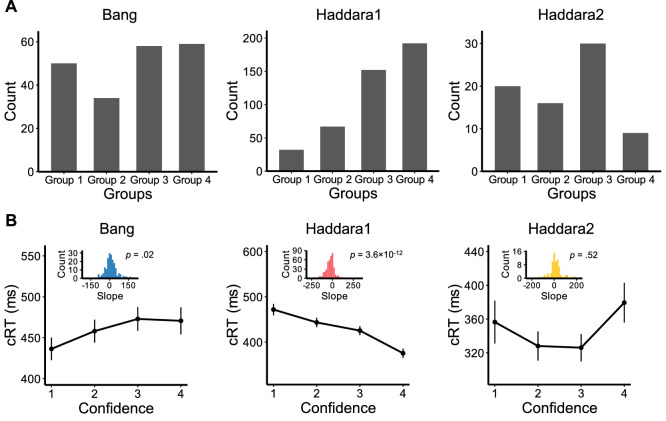
cRT–confidence relationship at the population level. (**A**) Number of subjects in each data set who used a specific confidence rating most frequently. “Group k” consists of all subjects for whom k is the most frequently chosen confidence rating. In Haddara1, most subjects used high confidence levels as their most frequent responses. This pattern is not present in Bang or Haddara2. (**B**) Average cRT for each confidence level. As can be seen in the figure, cRT decreases monotonically for higher confidence levels in Haddara1 but not in Bang or Haddara2. Insets are the histograms of slopes for different subjects. Error bars depict *SEM*.

Having strongly supported Hypothesis 2, we examined what that hypothesis predicts regarding the overall cRT–confidence relationship when all subjects are considered separately (i.e., the standard analysis in the literature; Moran et al., 2015; Pleskac & Busemeyer, 2010). According to Hypothesis 2, given that different subgroups show different directions of the cRT–confidence relationship, the direction of the relationship in the whole group would be driven by the most numerous subgroup. This is exactly what we found. In one data set (Haddara1), most subjects had a bias toward high confidence responses (Figure 4A), which should result in a negative overall relationship between cRT and confidence in the whole group. Indeed, we found a strong negative correlation between cRT and confidence at the population level in Haddara1 (slope = −30.95, 95% CI [−39.41, −22.47], *t*(417.24) = −7.16, *p* = 3.6 × 10^−12^, Cohen's *d* = −.35, BF10 = 1.5 × 10^9^; Figure 4B). However, the other two data sets (Haddara2 and Bang) featured relatively more balanced subgroup sizes (Figure 4A), which should result in much weaker overall relationships between cRT and confidence in the whole group. Indeed, we found no significant correlation between cRT and confidence at the population level in Haddara2 (slope = 6.20, 95% CI [−12.95, 25.36], *t*(74.52) = .64, *p* = 0.52, Cohen's *d* = .07, BF10 = .15; Figure 4B) and a slightly positive correlation in Bang (slope = 11.78, 95% CI [1.88, 21.68], *t*(186.92) = 2.34, *p* = 0.02, Cohen's *d* = .17, BF10 = 1.16). These results suggest that previous results of the population-level negative cRT–confidence relationship were likely due to most subjects having high confidence in those data sets. Indeed, this type of bias is clearly present in the Moran et al. (2015) data set (see Figure 4 in that article) and in the Herregods et al. (2023) data set (see Figure 7 in that article). These results demonstrate that the group-level cRT–confidence relationship is not fixed and depends on the overall level of bias toward low- or high-confidence responses in each data set.

### cRT–accuracy relationship

**Figure 5.**
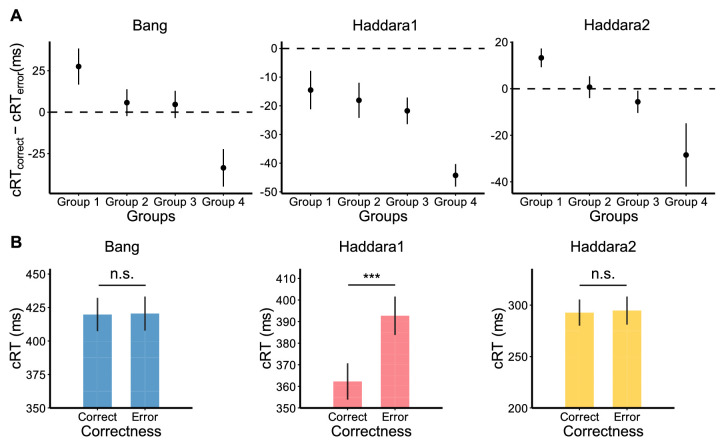
The cRT–accuracy relationship is driven by the most frequently chosen confidence rating. (**A**) The cRT–accuracy relationship, quantified as cRT_correct_ – cRT_error_, for each of the groups formed based on the most frequent confidence rating. (**B**) cRT for correct and error trials. As with the confidence results, correct trials were associated with lower cRTs in Haddara1 but not in Bang or Haddara2. Error bars show *SEM*. ***, *p* < 0.001; n.s., not significant.

Having shown that the cRT–confidence relationship is largely driven by the bias toward low- or high-confidence responses, we further examined whether the cRT–accuracy relationship is also driven by the same bias. Similar to the cRT–confidence relationship in Figure 3B, we found that cRT difference between correct and error trials (cRT_correct_ – cRT_error_) became smaller for the groups for which the most frequent confidence rating was higher (Bang: slope = −18.80, *t*(199) = −4.24, *p* = 3.4 × 10^−5^, Cohen's *d* = −.30; Haddara1: slope = −11.89, *t*(441) = −4.30, *p* = 2.1 × 10^−5^, Cohen's *d* = −.20; Haddara2: slope = −11.80, *t*(73) = −4.11, *p* = 1.0 × 10^−4^, Cohen's *d* = −.48; Figure 5A). In addition, similarly to the group-level cRT–confidence relationship (Figure 4B), we found that cRT was lower for correct compared with error trials in Haddara 1 (*t*(442) = −11.67, *p* = 1.3 × 10^−27^, Cohen's *d* = −.55, BF_10_ = 2.2 × 10^24^; Figure 5B) but not in the other two data sets (Bang: *t*(200) = −.13, *p* = 0.90, Cohen's *d* = −.009, BF_10_ = .07; Haddara2: *t*(74) = −.62, *p* = 0.54, Cohen's *d* = −.07, BF_10_ = .15). These results show that just as with the cRT–confidence relationship, the cRT–accuracy relationship is driven by each subject's confidence bias (i.e., the frequency with which they choose each confidence rating).

### RT–cRT relationship

**Figure 6.**
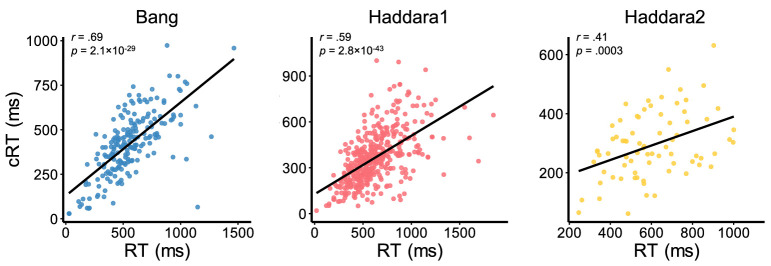
Correlations between RT and cRT. Scatterplots showing the across-subject association between mean cRT and mean RT for each of the three data sets. Each dot corresponds to one subject. Diagonal lines indicate best-fitting regressions.

Finally, we examined the correlations between RT and cRT to test whether the overall speed in decision and confidence responses may be related. Indeed, we found strong across-subject correlations between RT and cRT (Bang: *r* = .69, *p* = 2.1 × 10^−29^, BF_10_ = 1.5 × 10^26^; Haddara1: *r* = .59, *p* = 2.8 × 10^−43^, BF_10_ = 7.5 × 10^39^; Haddara2: *r* = .41, *p* = 3.0 × 10^−4^, BF_10_ = 76.57; Figure 6). These results suggest that the same factor contributes to response speed for both the decision and the confidence rating.

## Discussion

We set to adjudicate between two competing hypotheses regarding cRT: Hypothesis 1, which proposes that high-confidence responses are inherently made faster regardless of which confidence response is the most frequent, and Hypothesis 2, which proposes that the most frequent confidence responses are inherently made faster regardless of whether they are high or low. Several previous studies found a negative cRT–confidence relationship in the group as a whole (Herregods et al., 2023; Moran et al., 2015; Pleskac & Busemeyer, 2010). The authors interpreted these results as evidence for Hypothesis 1 and used them to motivate models where confidence is based on a postdecision evidence accumulation process. Here we compared the predictions of the two hypotheses using three large data sets from the Confidence Database. We found that the most frequent confidence responses were made faster regardless of whether confidence was high or low, supporting Hypothesis 2 and rejecting Hypothesis 1. These findings reveal the factors driving confidence response times and challenge several postdecisional models of confidence.

To be clear, our results do not falsify all postdecisional models of confidence. Three prominent postdecisional models—the 2DSD model with optional stopping (Pleskac & Busemeyer, 2010), the collapsing confidence boundary model (Moran et al., 2015), and the recent Herregods et al. model (Herregods et al., 2023)—postulate that cRT is intrinsically negatively related to confidence (Hypothesis 1 above). Therefore, by falsifying Hypothesis 1, our results directly challenge these models. However, there are other postdecisional confidence models that assume constant postdecisional evidence accumulation time (Pleskac & Busemeyer, 2010). For example, unlike the 2DSD model with optional stopping, which allows interjudgment time to vary between trials, the main 2DSD model just treats the interjudgment time as a constant exogenous parameter in the model (Pleskac & Busemeyer, 2010). While the original versions of these models are also challenged by the current results (because these models do not predict that cRT would vary with the frequency of the confidence rating), it should be possible to augment these models with extra parameters that make the interjudgment time dependent on the frequency of the confidence response.

We also want to clarify that our results do not challenge the notion that information arriving after the decision can be used to influence the eventual confidence rating. There is considerable behavioral and neural evidence that confidence judgments can indeed be influenced by information or processing that occurs after the initial decision has been made (Boldt & Yeung, 2015; Desender et al., 2021; Pereira et al., 2020). It is important to note, however, that while many models do not explicitly incorporate the possible influences of information coming after the decision, virtually all existing models of metacognition (Fleming & Daw, 2017; Green & Swets, 1966; Jang et al., 2012; Maniscalco & Lau, 2016; Rausch et al., 2018; Shekhar & Rahnev, 2021) can be extended to do so if desired.

Why are the most frequent confidence ratings made faster? One possible mechanism is that the motor system is able to execute more frequent actions faster (Katzner & Miller, 2012; Mattes et al., 2002; Miller, 1998; Näätänen, 1971). Specifically, low response frequency is thought to lead to poor motor preparation, which results in slower responses (Näätänen, 1971). The motor influence on response speed has been confirmed by the finding that lateralized readiness potential (an electrophysiologic indicator of motor preparation) is larger for more frequent responses (Eimer, 1998; Miller, 1998) and by showing that the correlation cannot be explained by properties of external stimuli, such as the frequency of different stimuli (Katzner & Miller, 2012; Mattes et al., 2002). Our findings extend this previous work to confidence judgments and suggest that motor influences might underlie the relationship between the response frequency and cRT.

Although our work here focused on cRT, our findings raise questions regarding potential influences for decision RTs too. Indeed, similar to the results here, it is commonly found that subjects are faster for the choices they give more frequently (de Lange et al., 2013; Rahnev et al., 2011). However, such contingencies are usually assumed to arise exclusively from the evidence accumulation process (e.g., as a consequence of a biased starting point of the accumulation) (Brown & Heathcote, 2008; Ratcliff & McKoon, 2008; Ratcliff & Smith, 2004). Indeed, classical evidence accumulation models of decision-making such as drift-diffusion model (DDM) usually decompose RTs into decision and nondecision time and assume that the nondecision time is constant across all choices regardless of differences in the frequency of different choices (Ratcliff & McKoon, 2008; Ratcliff & Smith, 2004). Our findings cast doubt on this assumption and suggest that more frequent choices have lower RTs not only because of effects related to the decision process (e.g., starting point or drift rate bias) but also due to nondecision times that are faster for more frequent choices.

In conclusion, our work shows that cRT and confidence are not intrinsically related, and instead, cRT is simply lower for the most frequent confidence responses. These results strongly challenge several postdecision evidence accumulation models, constrain future theories of confidence generation, and suggest the need for more careful examination of standard accumulation-to-bound theories of perceptual decision-making.

## Supplementary Material

Supplement 1
