# Supplementary material for: Confidence response times: Challenging postdecisional models of confidence
Source: J Vis. 2023 Jul 14;23(7):11. doi: 10.1167/jov.23.7.11 (PMC10353741; doi:10.1167/jov.23.7.11)
Supplement: Supplement 1 [file jovi-23-7-11_s001.pdf]

Supplementary Materials for "Confidence response times: Challenging post-decisional models of confidence"

Sixing Chen[1], Dobromir Rahnev[2]
[1]School of Psychological and Cognitive Sciences, Peking University, Beijing, China
[2]School of Psychology, Georgia Institute of Technology, Atlanta, GA, USA

Supplementary Methods

To examine whether the results from the main paper replicate in a larger number of datasets, we relaxed criterion 3 used for the selection of datasets. Specifically, we changed that criterion so that we would select datasets with at least 30 subjects (instead of 75 subjects) who each completed at least 150 trials per task (instead of 200). This resulted in selecting 5 additional datasets. However, two of the datasets could not be used and had to be excluded. Specifically, the Siedlecka_2021 dataset has an experimental design in which subjects could choose not to respond on some trials, and the Xu_2019_Exp1 dataset has most subjects (27/30) used the maximum confidence level of 4 most frequently. These features made it impossible to run our analyses on these two datasets. Thus, we report results from the other 3 additional datasets: Maniscalco_2017_expt1, Maniscalco_2017_expt2, and Yeon_unpub_Exp2 (which we named Maniscalco1, Maniscalco2, and Yeon). The results of these datasets (Figures S1-3) are in line with the results of the datasets that we analyzed in the main paper.

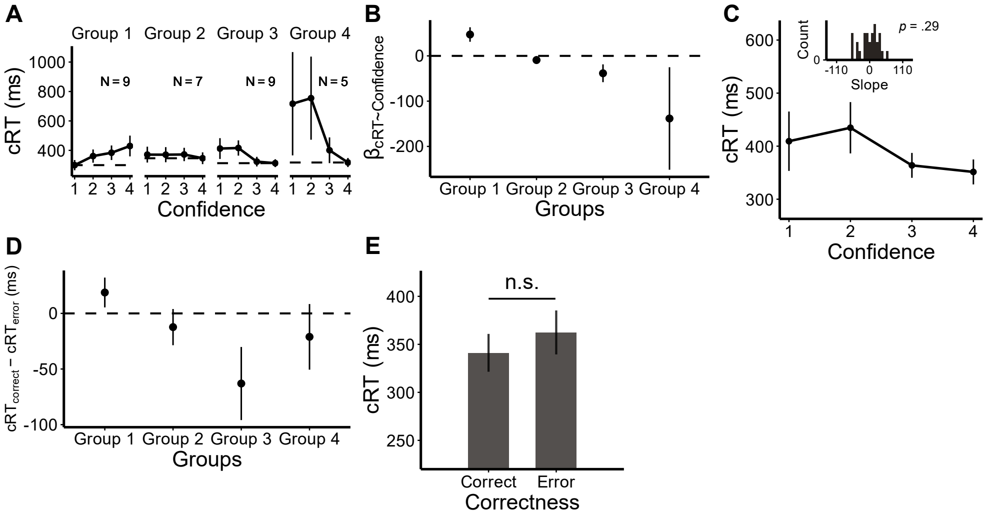

Figure S1. Analyses of Maniscalco1 dataset. The name of the Maniscalco1 dataset in the Confidence Database is "Maniscalco_2017_expt1" (Maniscalco et al., 2017). In the task, circular patches of white noise were presented to the left and right of fixation. A sinusoidal grating was embedded in either the left or right patch of noise. Subjects indicated which patch contained the grating, left or right, with a keypress. After that, subjects rated confidence on a 4-point scale. 30 subjects each completed 1000 trials. More details about the dataset can be found in the original publication. In the dataset, both cRT-confidence and cRT-accuracy relationships are driven by the response frequency, and are not significant at the population level. (A) cRT for each confidence rating for each group formed based on the most frequent confidence rating. In line with Hypothesis 2, cRT was lowest for the most frequent confidence rating for groups 1 and 4, though the effect was less clear in groups 2 and 3, presumably due to the smaller power for this dataset. (B) Analyzing all groups together showed that the slope of the cRT-confidence relationship (i.e., $\beta_{\text{cRT}\sim\text{Confidence}}$) decreases for the groups where the most frequent confidence rating is higher (slope = -54.36, $t(27) = -3.48$, $p = .002$, Cohen's $d = -.66$), indicating that the cRT-confidence relationship is driven by the response frequency. (C) There is no significant cRT-confidence relationship at the population level (slope = -23.97, 95% CI = [-68.07, 20.14], $t(29.02) = -1.08$, $p = .29$, Cohen's $d = -.20$, BF10 = .33), consistent with the lack of clear bias in the group towards either high or low confidence responses. (D) The cRT difference between correct and error trials generally decreases for the groups where the most frequent confidence rating is higher (slope = -23.54, $t(27) = -1.95$, $p = .06$, Cohen's $d = -.37$). The effect was not significance presumably due to the limited power in this experiment. (E) There is no significant cRT-accuracy relationship at the population level ($t(28) = -1.48$, $p = .15$, Cohen's $d = -.27$, BF10 = .52), consistent with the lack of clear bias in the group towards either high or low confidence responses. Error bars show *SEM*. ***, $p < 0.001$; *, $p < .05$; n.s., not significant.

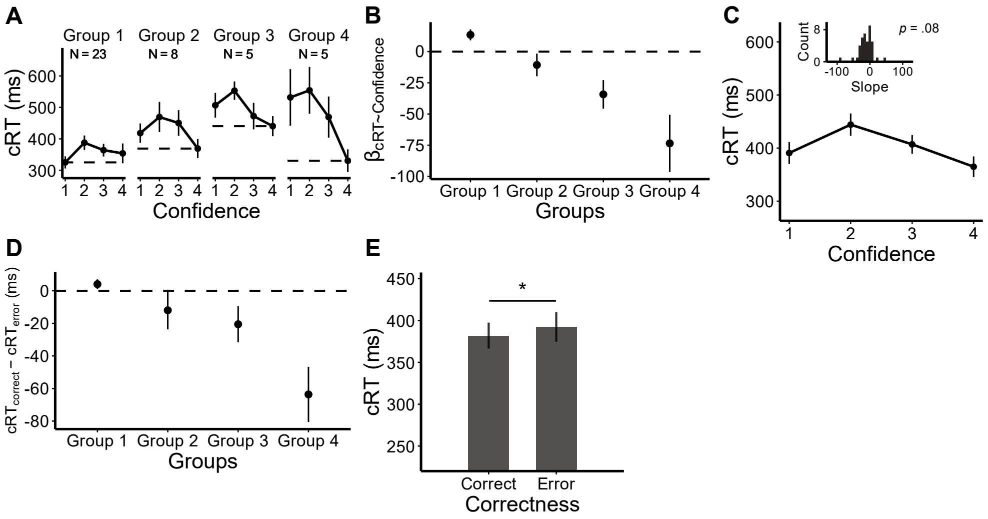

Figure S2. Analyses of Maniscalco2 dataset. The name of the Maniscalco2 dataset in the Confidence Database is "Maniscalco_2017_expt2" (Maniscalco et al., 2017). The experimental design of the Maniscalco2 dataset is identical to the Maniscalco1 dataset. 41 subjects each completed 510 trials. More details about the dataset can be found in the original publication. In the dataset, both cRT-confidence and cRT-accuracy relationships are driven by the response frequency. The cRT-confidence relationship is not significant at the population level. For the cRT-accuracy relationship, cRT is significantly lower in correct trials than in error trials. (A) cRT for each confidence rating for each group formed based on the most frequent confidence rating. In line with Hypothesis 2, cRT was lowest for the most frequent confidence rating for groups1 and 4, though the effect was not present in groups 2 and 3, presumably due to the limited subject number in this experiment. (B) Analyzing all groups together showed that the slope of the cRT-confidence relationship (i.e., $\beta_{\text{cRT\sim Confidence}}$) decreases for the groups where the most frequent confidence rating is higher (slope = -27.51, $t(39) = -7.00$, $p = 2.2\times10^{-8}$, Cohen's d = -1.11), indicating that the cRT-confidence relationship is driven by the response frequency. (C) There is no significant cRT-confidence relationship at the population level (slope = -11.50, %95 CI = [-24.24, 1.28], $t(40.14) = -1.79$, $p = .08$, Cohen's d = -.28, BF10 = .72), consistent with the lack of clear bias in the group towards either high or low confidence responses. (D) The cRT difference between correct and error trials decreases for the groups where the most frequent confidence rating is higher (slope = -19.70, $t(39) = -5.69$, $p = 1.4\times10^{-6}$, Cohen's d = -.90), indicating that the cRT-accuracy relationship is driven by the response frequency. (E) cRT was slightly lower in correct than in error trials ($t(40) = -2.10$, $p = .04$, Cohen's d = -.33, BF10 = 1.21). Error bars show *SEM*. ***, $p < 0.001$; *, $p < .05$; n.s., not significant.

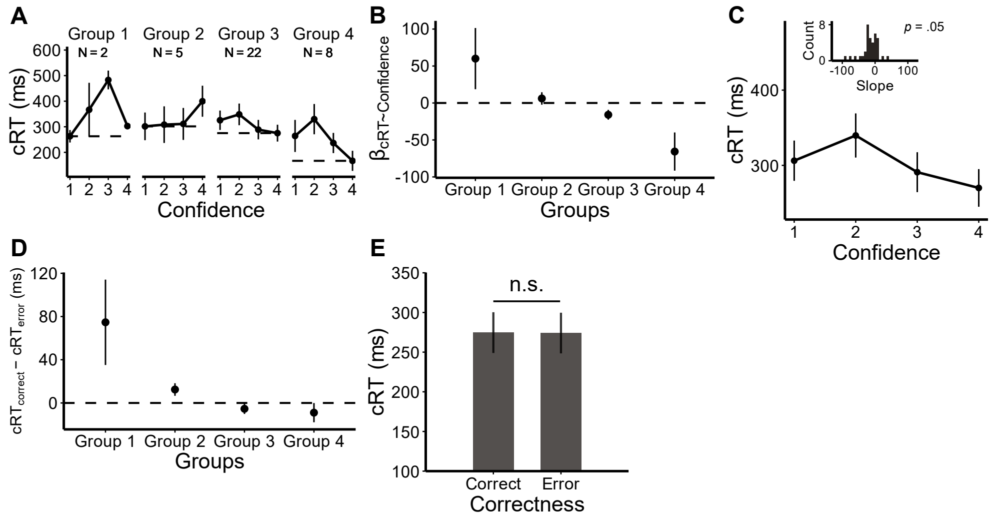

Figure S3. Analyses of Yeon dataset. The name of the Yeon dataset in the Confidence Database is "Yeon_unpub_Exp2". In the task, a large number of white dots were moving in random directions while a certain proportion of dots were moving toward either left or right direction. Subjects indicated the direction of the coherently moving dots. After that, subjects rated confidence on a 4-point scale. 37 subjects each completed 800 trials. More details about the dataset can be found in https://osf.io/g7ary. In the dataset, both cRT-confidence and cRT-accuracy relationships are driven by the response frequency, and are not significant at the population level. (A) cRT for each confidence rating for each group formed based on the most frequent confidence rating. In line with Hypothesis 2, cRT was lowest for the most frequent confidence rating for groups 1 and 4, though the effect was less clear in groups 2 and 3, presumably due to the smaller power for this dataset. (B) Analyzing all groups together showed that the slope of the cRT-confidence relationship (i.e., $\beta_{cRT\sim Confidence}$) decreases for the groups where the most frequent confidence rating is higher (slope = -38.90, $t(35) = -4.17$, $p = 1.9\times10^{-4}$, Cohen's $d = -.69$), indicating that the cRT-confidence relationship is driven by the response frequency. (C) There is no significant cRT-confidence relationship at the population level (slope = -15.16, %95 CI = [-30.39, -.23], $t(28.01) = -2.01$, $p = .05$, Cohen's $d = -.37$, BF10 = 1.13), consistent with the lack of clear bias in the group towards either high or low confidence responses. (D) The cRT difference between correct and error trials decreases for the groups where the most frequent confidence rating is higher (slope = -20.60, $t(35) = -3.68$, $p = 7.8\times10^{-4}$, Cohen's $d = -.61$), indicating that the cRT-accuracy relationship is driven by the response frequency. (E) There is no significant cRT-accuracy relationship at the population level ($t(36) = .12$, $p = .91$, Cohen's $d = .02$, BF10 = .18), consistent with the lack of clear bias in the group towards either high or low confidence responses. Error bars show *SEM*. ***, $p < 0.001$; *, $p < .05$; n.s., not significant.

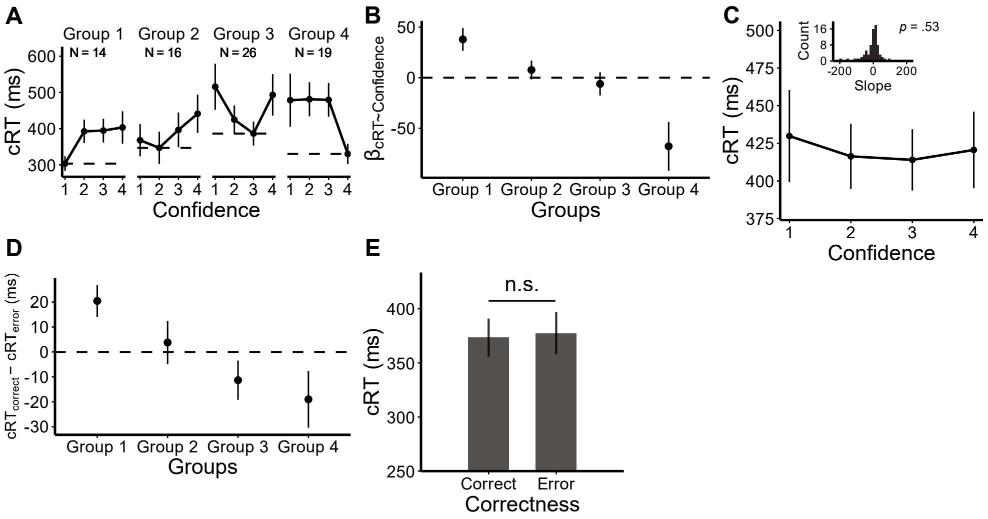

Figure S4. Analyses of Day 1 of Haddara2 dataset. The results of Day 1 are consistent with the results of all 7 days reported in the main paper. (A) cRT for each confidence rating for each group formed based on the most frequent confidence rating. In line with the results of all 7 days, cRT was lowest for the most frequent confidence rating. (B) Analyzing all groups together showed that the slope of the cRT-confidence relationship (i.e., $\beta_{\text{cRT~Confidence}}$) decreases for the groups where the most frequent confidence rating is higher (slope = -33.18, $t(72) = -4.43$, $p = 3.2 \times 10^{-5}$, Cohen's d = -.52), indicating that the cRT-confidence relationship is driven by the response frequency. The results are consistent with the results of all 7 days. (C) There is no cRT-confidence relationship at the population level (slope = -5.88, %95 CI = [-24.30, 12.51], $t(69.47) = -.63$, $p = .53$, Cohen's d = -.08, BF10 = .16), consistent with the lack of clear bias in the group towards either high or low confidence responses. The results are in accordance with the results of all 7 days. (D) The cRT difference between correct and error trials decreases for the groups where the most frequent confidence rating is higher (slope = -13.26, $t(73) = -3.10$, $p = .003$, Cohen's d = -.36), indicating that the cRT-accuracy relationship is driven by the response frequency. The results are consistent with the results of all 7 days. (E) There is no significant cRT-accuracy relationship at the population level ($t(74) = -.86$, $p = .39$, Cohen's d = -.10, BF10 = .18), consistent with the lack of clear bias in the group towards either high or low confidence responses. The results are consistent with the results of all 7 days. Error bars show *SEM*. ***, $p < 0.001$; *, $p < .05$; n.s., not significant.

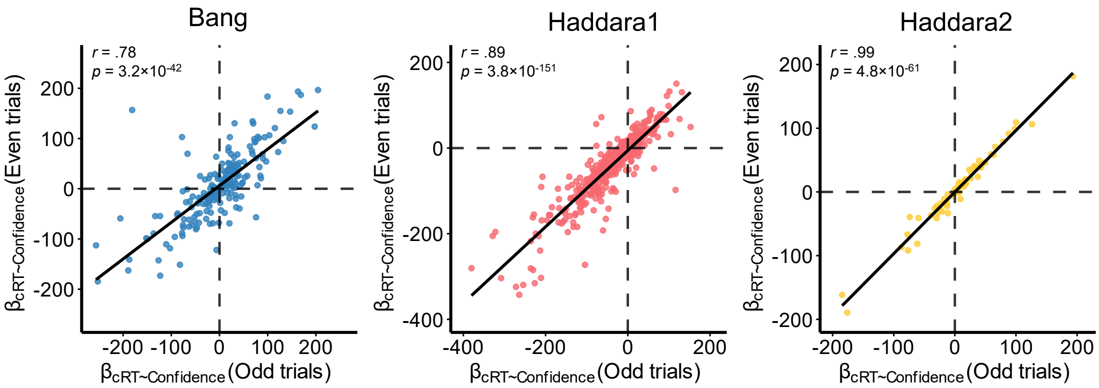

Figure S5. cRT-confidence relationship at the individual level. We computed the cRT-confidence relationship separately based on the odd and even trials for each subject, and then correlated the strengths of the relationship across subjects. We found very high correlations between odd and even trials for the strength of the cRT-confidence relationship, quantified as $\beta_{\text{cRT}\sim\text{Confidence}}$ (Bang: $r = .78$, $p = 3.2\times10^{-42}$; Haddara1: $r = .89$, $p = 3.8\times10^{-151}$, bootstrapped $r = .84$; Haddara2: $r = .99$, $p = 4.8\times10^{-61}$, bootstrapped $r = .98$). Importantly, there were high proportions of subjects with consistently positive or consistently negative cRT-confidence relationship, suggesting the existence of substantial and reliable individual differences in the cRT-confidence relationship. In all computations, cRTs were in ms. Each dot corresponds to one subject. Solid lines indicate best-fitting regressions.

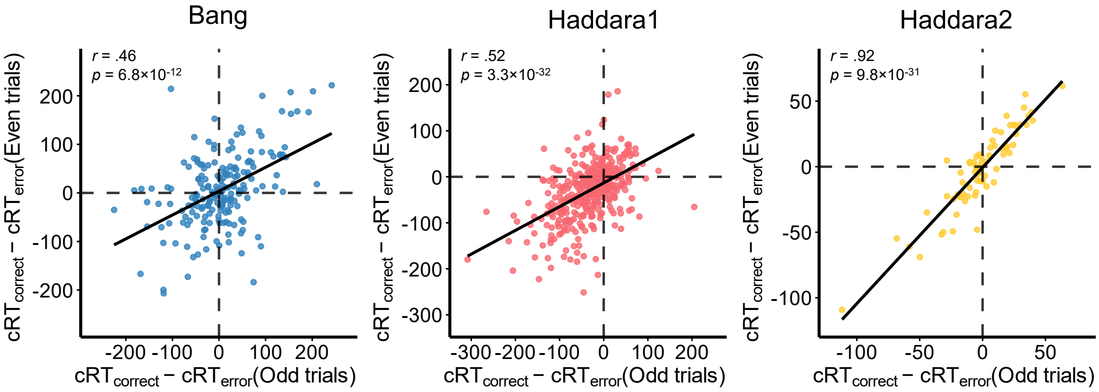

Figure S6. cRT-accuracy relationship at the individual level. We computed the cRT-accuracy relationship separately based on the odd and even trials for each subject, and then correlated the strengths of the relationship across subjects. We found very high correlations between odd and even trials for the cRT-accuracy relationship, quantified as $cRT_{correct} - cRT_{error}$ (Bang: $r = .46$, $p = 6.8 \times 10^{-12}$, bootstrapped $r = .40$; Haddara1: $r = .52$, $p = 3.3 \times 10^{-32}$, bootstrapped $r = .54$; Haddara2: $r = .92$, $p = 9.8 \times 10^{-31}$, bootstrapped $r = .88$). Crucially, there were high proportions of subjects with consistently positive or consistently negative cRT-accuracy relationships. In all computations, cRTs were in ms. Each dot corresponds to one subject. Solid lines indicate best-fitting regressions.
